# Treatment Outcomes and Survival in Hypercalcemia of Malignancy: A Grave Metabolic Emergency

**DOI:** 10.7759/cureus.35783

**Published:** 2023-03-05

**Authors:** Sweety Gupta, Aviral Rastogi, Pragya Singh, Atokali Chophy, Ravi Roushan, Ajay S Krishnan, Deepa Joseph, Bela Goyal, Amit Gupta, Manoj Gupta

**Affiliations:** 1 Department of Radiation Oncology, All India Institute of Medical Sciences, Rishikesh, IND; 2 Department of Radiation Oncology, Tata Memorial Hospital, Varanasi, IND; 3 Department of Biochemistry, All India Institute of Medical Sciences, Rishikesh, IND; 4 Department of Surgery, All India Institute of Medical Sciences, Rishikesh, IND

**Keywords:** hypercalcemia of malignancy, prognosis, survival, bisphosphonates, calcium, hypercalcemia

## Abstract

Background: Management of hypercalcemia is based on the manifestation of symptoms and serum calcium levels. It is considered an oncological emergency; therefore, management has to be done on an urgent basis.

Aim: In the present study, we analyzed the clinicopathological profile, treatment, and outcome of patients with hypercalcemia in solid malignancies at our institute.

Methods: We retrospectively analyzed the medical records of patients diagnosed with cancer and admitted to the department of radiation oncology with hypercalcemia. The parameters studied were age, gender, performance status, date of diagnosis, the primary site of cancer, stage, histopathology, time of presentation of hypercalcemia since initial cancer diagnosis, clinical symptoms, parathyroid hormone levels, liver and renal function tests, bone metastases, management, outcome, and present status.

Results: In the present study, 47 patients of hypercalcemia from various solid malignancies were admitted during the study period between 1st January 2018 and 30th April 2022. Head and neck cancer (14, 29.7%) was the most common site of the primary malignancy. Twelve patients had incidental hypercalcemia and were asymptomatic. Management of hypercalcemia included intravenous saline hydration, bisphosphonates, and supportive medication. At the time of analysis, 17 patients were lost to follow-up, 23 patients died, and seven were alive and on follow-up. Median survival was 68.0 days (95% CI: 1.7-134.3 days).

Conclusion: Hypercalcemia of malignancy is considered a metabolic oncological emergency and requires urgent and aggressive management. It gets complicated by a deranged kidney function test. Despite available treatment, it portends an abysmal prognosis.

## Introduction

Hypercalcemia is defined as an increase in the serum calcium level above the upper limit of normal. Hypercalcemia of malignancy (HOM) occurs in approximately 44.1% of all cancer patients [1]. It is more common in advanced stages of malignancy [2]. The more common cancers associated with HOM are lung, breast, multiple myeloma, non-Hodgkin’s lymphoma, squamous cell cancer, and ovary [3]. The primary pathophysiologic mechanism of HOM in 80% of patients is parathyroid hormone-related protein (PTHrP) production. PTHrP acts on osteoblasts and activates osteoclasts, thus increasing bone resorption and serum calcium levels. Of the cases, 20% occur due to osteolytic bone metastases causing calcium release into the blood. Other mechanisms are an ectopic activity of 1-alpha-hydroxylase leading to the formation of 1,25-dihydroxycholecalciferol and ectopic production of parathyroid hormone (PTH) [4].

The clinical symptoms are based on serum calcium levels and the rapid development of hypercalcemia. Symptoms include neurocognitive, gastrointestinal, and renal. The cardiovascular system is also affected due to raised calcium levels, and an electrocardiogram (ECG) shows a short QT interval [5].

Hypercalcemia is classified as mild, moderate, and severe according to serum calcium levels <12 mg/dl, 12-14mg/dl, and above 14mg/dl, respectively. Management of hypercalcemia is based on the manifestation of symptoms and serum calcium levels. HOM is considered an oncologic emergency; therefore, management has to be done on an urgent basis. The action aims to lower serum calcium levels and treat the underlying cause. The management for hypercalcemia includes intravenous hydration, calcitonin, bisphosphonates, denosumab, gallium nitrate, and prednisolone, and in cases of chronic kidney diseases, the patient may require hemodialysis. Although bisphosphonates and receptor activators for nuclear factor kappa B ligand (RANKL) therapy are adequate to attain normocalcemia, the overall prognosis remains grave. Around 50% of patients die within 30 days, and up to 75% die within three months of diagnosis [6].

Therapies are available for short and long-term management of HOM, but the order in which they should be administered depending on severity is still not clear.

To the best of our knowledge, there is a paucity of studies that have evaluated the outcome of HOM in the Indian scenario. Therefore, we conducted the study to analyze the clinicopathological profile, treatment, and outcome of patients with hypercalcemia in solid malignancies and compare the results with the literature.

## Materials and methods

Patient selection

We retrospectively analyzed the medical records of patients diagnosed with cancer and admitted to the department of radiation oncology with hypercalcemia between 1st January 2018 and 30th April 2022. Patients with a cancer diagnosis and age ≥ 18 years were included, and patients with hematological malignancies, primary hyperparathyroidism, end-stage renal disease, and decompensated liver disease were excluded. Renal injury and tumor lysis syndrome were excluded prior to inclusion in the study. Approval was taken from the Institutional Ethics Committee, All India Institute of Medical Sciences, Rishikesh (letter no: AIIMS/IEC/22/272; dated 27/05/2022).

Study parameters

The parameters studied were age, gender, performance status, date of diagnosis, the primary site of cancer, stage, histopathology, time of presentation of hypercalcemia since initial cancer diagnosis, clinical symptoms, PTH levels, liver and renal function tests, bone metastases, management, outcome, and present status. Diagnosis of HOM was established if corrected serum calcium was ≥10.3 mg/dl, along with a low or borderline normal value of serum intact PTH levels, normal serum vitamin D levels, and a normal thyroid function test. Serum calcium level grading was done as follows: normal: 8.5-10.3 mg/dl; mild: 10.4-11.9 mg/dl; moderate: 12-14 mg/dl; severe: >14 mg/dl. Correction of calcium for albumin level was done in patients with abnormal (both low and elevated levels) albumin and calculated by the following formula: calcium (mg/dl) + 0.8 (4.0 − patient's albumin level). Creatinine clearance was calculated by the Cockcroft-Gault formula: creatinine clearance (ml/min) = (140 − age) x lean body weight (in kg) x 0.85 (if female)/serum creatinine (mg/dl) x 72. A dose adjustment of zoledronic acid was made if creatinine clearance was <50 ml/min.

Statistical analysis

All data were recorded and analyzed. Categorical variables were described with frequencies and percentages. Continuous variables were defined using either means with standard deviations or medians with appropriate percentiles. The primary outcome was overall survival (inferred from hospital records, death certificates, and telephonic information from caregivers). Overall survival was defined as the date of first documentation of hypercalcemia event until death. Patients who were lost to follow-up were excluded from the survival analysis.

## Results

Patient characteristics

In the present study, 47 patients of hypercalcemia from various solid malignancies were admitted during the study period. Age ranged from 26 to 78 years (mean: 48.3). The most common age group was 41-50 years. Males and females were 1.04:1. Bone metastases were present in 18 (38.2%) patients. The time of occurrence of hypercalcemia varied from six days to 33 months after the initial primary diagnosis. Twenty-one patients presented with hypercalcemia within three months of diagnosis and were on treatment. Patient characteristics and biochemical parameters are mentioned in Table 1.

**Table 1:** Clinical and biochemical parameters of patients with hypercalcemia

Variables	Options	Number (%)
Age (in years)	Mean (range)	48.7 (26-78) years
	≥18-30	3 (6.3)
	31-40	10 (21.2)
	41-50	16 (34.0)
	51-60	9 (19.1)
	61-70	7 (14.8)
	>70	2 (4.2)
Gender	Male	23 (48.9)
	Female	24 (51.0)
Histology	Adenocarcinoma	6 (1.2)
	Squamous	26 (55.3)
	Ductal carcinoma	11 (23.4)
	Others	4 (8.5)
Stage	III	3 (6.3)
	IV	44 (93.6)
Bone metastases	Yes	18 (38.2)
	No	29 (61.7)
Time for the occurrence of hypercalcemia after diagnosis	Mean (range)	7(1-39) months
	<3 months	21 (44.0)
	3 to 6 months	11 (23.0)
	6 to 12 months	05 (11.0)
	1 to 2 years	06 (13.0)
	>2 years	04 (9.0)
Grading of hypercalcemia	Mean	13.1 (10.6-16.5) mg/dl
	Mild: 10.4-11.9 mg/dl	14 (29.8)
	Moderate:12-14 mg/dl	18 (38.3)
	Severe: >14 mg/dl	15 (31.9)
Deranged renal function (urea > 43 mg/dl; creatinine > 1.3 mg/dl)	Yes	17 (36.2)
	No	30 (63.8)
Recovery in days	Mean ± SD	9.61 ± 7.31
	Within 4 days	08 (17.0)
	4 to 10 days	25 (53.0)
	More than 10 days	14 (30.0)
*Others: glioblastoma multiforme, sacral giant cell tumor, unknown primary with secondary neck		

**Figure 1:**
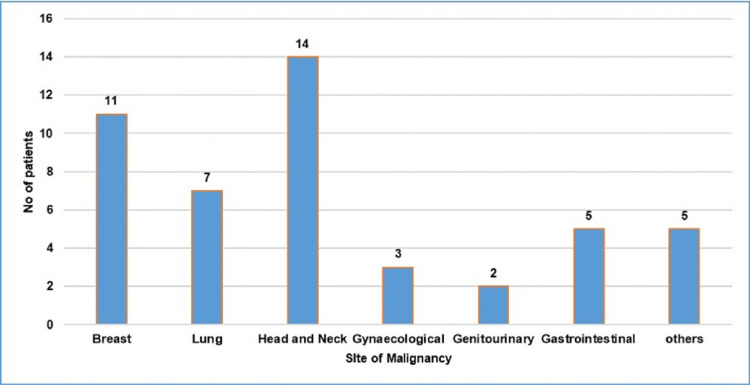
Distribution of patients with hypercalcemia according to site

The most common site of primary malignancy was head and neck cancer in 14 (29.7%) patients, followed by breast cancer in 11 (23.4%), and lung cancer in seven (14.8%) patients. One patient with glioblastoma and sacral giant cell tumor had hypercalcemia (Figure 1).

Clinical presentation and management

Most patients' clinical symptoms were related to a primary disease or non-specific. Nine patients had incidental hypercalcemia on investigations and were asymptomatic. Most patients with incidental hypercalcemia diagnosis had mild or moderate grades, with only one patient having more than 14 mg/dl calcium levels. Seventeen (36.2%) patients had deranged renal function tests. Management of hypercalcemia included intravenous saline hydration, bisphosphonates, and supportive medication. In patients with deranged kidney function tests, the dose of zoledronic acid was reduced accordingly. The intent of treatment was palliative chemotherapy and radiotherapy in 41 patients, adjuvant and radical in one each, and neoadjuvant chemotherapy in two patients.

Follow-up and survival

**Figure 2:**
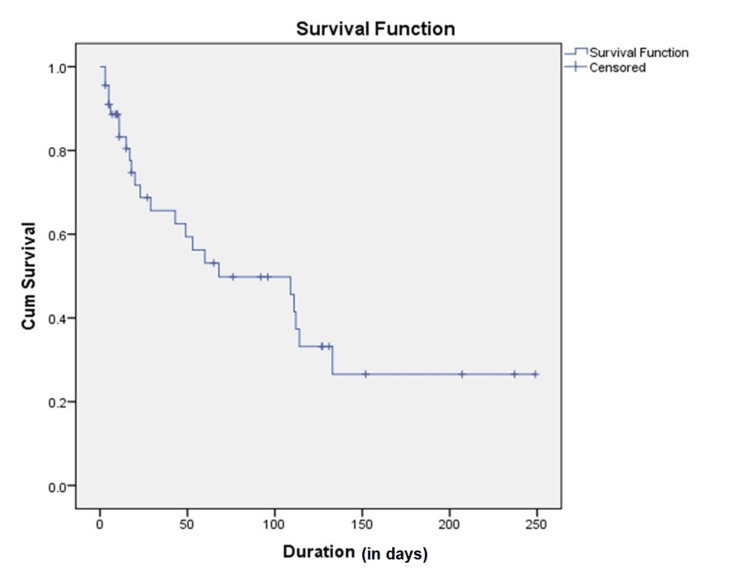
Kaplan-Meier survival graph of patients with hypercalcemia of malignancy

Duration of admission ranged from three to 28 days (mean: 8.5 days). Eight (17%) patients expired during admission. Out of eight patients, seven died within 30 days of diagnosis of hypercalcemia. The reason for death was complications of hypercalcemia in eight patients. Fifteen patients died due to disease progression during the course of treatment. The mean days for recovery from hypercalcemia was 9.6 days, and most patients stayed (53%) for four to 10 days of hospitalization, and 14 (30%) patients recovered after 10 days. Two patients had a reoccurrence of hypercalcemia after management. The mean duration of follow-up was 8.3 (range: 0-42) months. At the time of analysis, 17 patients were lost to follow-up, 23 patients died, and seven were alive and on follow-up. Median survival (Figure 2) was 68 days (95% CI: 1.7-134.3 days).

## Discussion

Hypercalcemia was initially described in 1921 and is seen in around 40% of patients with malignancy during their disease. The most common malignancy associated with HOM is multiple myeloma, and in solid tumors, breast and renal cell carcinoma are followed by squamous cell cancer of any origin [7,8]. Malignancies rarely associated with HOM are brain tumors and prostate, gastric, and colorectal cancers [9]. In the present study, head and neck cancer subsite oral cavity was the most common malignancy, and patients were in advanced stages, the most putative reason being squamous in origin. Breast cancer was the second most common malignancy. There was one patient with glioblastoma multiforme with hypercalcemia, though reported to be very rare in literature [9]. The most common pathogenesis of HOM is humoral due to the release of PTHrP secretion from tumor cells, followed by osteolytic bone metastases leading to hypercalcemia. In the present study, not all patients could be tested for PTH levels. Eighteen patients with hypercalcemia had osteolytic bone metastases.

Clinical symptoms of patients with hypercalcemia are non-specific and unrelated to etiology [10]. Symptoms depend on serum calcium levels and the rapidity with which it rises. Neurological symptoms include lethargy, confusion, and in severe cases, coma. Gastrointestinal symptoms include constipation, loss of appetite, and nausea. Cardiovascular symptoms include QT interval shortening on ECG [11]. In the present study, 12 patients were asymptomatic, whereas others had non-specific symptoms. Even non-specific symptoms of patients decreased once their serum calcium had normalized.

Management of HOM depends on the severity of hypercalcemia and underlying malignancy: intravenous hydration, bisphosphonates, steroids, and gallium nitrate. Newer therapies include denosumab and cinacalcet.

The goals of treating hypercalcemia are to increase the elimination of calcium from the extracellular fluid, reduce gastrointestinal (GI) absorption, and decrease bone resorption.

Patients with asymptomatic or mild symptomatic hypercalcemia (<12 mg/dl) do not require aggressive treatment. However, factors aggregating hypercalcemia should be avoided, including thiazide diuretics, lithium carbonate, volume depletion, prolonged inactivity, a high-calcium diet (>1000 mg/day), and calcium/vitamin D supplements. Adequate hydration (six to eight glasses of water per day) is recommended to minimize the risk of nephrolithiasis. HOM is considered an indicator of advanced stages of the disease and leads to organ failure and sepsis. Also, the median time to relapse is very short. Even if serum calcium is normalized, the prognosis remains poor and patients are candidates for the best supportive care. Further treatment depends mainly upon the etiology of hypercalcemia.

Sharp rise to moderate hypercalcemia may cause marked changes in the sensorium, requiring aggressive therapy. Treatment includes saline hydration and bisphosphonates. Immediate therapy aims to restore intravascular volume and promote calcium excretion in the urine with an infusion of 0.9% saline at twice the maintenance rate until any fluid deficit is replaced and diuresis is achieved (urine output ≥ 200-300 mL/h). Hemodialysis is the treatment of choice in patients with heart failure or renal insufficiency for rapid reduction of calcium levels. Bisphosphonates such as zoledronic acid, pamidronate, and alendronate are the drugs of choice for HOM as they inhibit osteoclastic activity.

Severe hypercalcemia therapy includes simultaneous administration of intravenous isotonic saline, subcutaneous calcitonin, and bisphosphonate (typically, IV zoledronic acid). The administration of calcitonin plus saline hydration substantially decreases serum calcium levels within 12-48 hours. The effect of bisphosphonate starts on the second to fourth day and provides a more sustained impact, thereby maintaining control of the hypercalcemia. To prevent a recurrence, follow-up therapy is recommended. In patients with HOM, underlying malignancy should be treated, and patients with bone metastases may require monthly zoledronic acid. Also, calcium and vitamin D intake should be restricted in such patients [11].

Denosumab is a monoclonal antibody approved for osteoporosis and for preventing skeletal-related events [12]. Also, it is safe in chronic kidney disease, as it is not metabolized through the kidney. Cinacalcet interacts with the calcium-sensing receptor located on parathyroid cells leading to the downregulation of PTH and subsequent decrement of serum calcium levels [13]. In the present study, patients were also treated as per the algorithm of management of HOM, treatment included intravenous saline hydration, bisphosphonates (zoledronic acid), and steroids.

A pooled analysis of two randomized trials was done by Major et al., and it was identified that calcium level decrement started within 48 hours of bisphosphonates administration, with normocalcemia achieved after 96 hours [14]. Ralston et al. and Fisken et al. have reported that median survival is one to three months after the diagnosis of HOM [15,16]. Vassilopoulou-Sellin et al. also said that only 25% of patients survived for one year [17]. In a study of 138 patients with humoral HOM, Donovan et al. reported that median survival was 52 days (21-132) [18]. In the present study, median survival was 68 days, comparable to previous studies. This suggests HOM itself portends a dismal prognosis despite hypercalcemia being corrected with medical measures.

HOM is considered an indicator of advanced stages of the disease and leads to organ failure and sepsis. Also, the median time to relapse is very short. Even if serum calcium is normalized, the prognosis remains poor and patients are candidates for best supportive care due to poor performance status.

Future research should focus on the comparison of bisphosphonates and denosumab in the treatment of hypercalcemia, rebound hypercalcemia, and the cost involved in the treatment, with any difference in survival.

Limitations of the present study were that it was a retrospective analysis, and parathormone levels were not measured in most patients due to logistic issues.

## Conclusions

HOM is considered a metabolic oncological emergency and requires urgent and aggressive management. The clinical symptoms are based on serum calcium levels and the rapidity of development of hypercalcemia. It gets complicated by a deranged kidney function test. Despite available treatment, it portends an abysmal prognosis. Despite the correction of hypercalcemia, median overall survival remained poor in our patients, suggesting the best supportive care could have been an option for these patients. Definitive management may be an option in treatment naïve patients with good performance status. Future research should focus on the comparison of bisphosphonates and denosumab in the treatment of hypercalcemia, rebound hypercalcemia, and the cost involved in the treatment, with any difference in survival.
